# Work Function Variations in Twisted Graphene Layers

**DOI:** 10.1038/s41598-018-19631-4

**Published:** 2018-01-31

**Authors:** Jeremy T. Robinson, James Culbertson, Morgann Berg, Taisuke Ohta

**Affiliations:** 1 Naval Research Laboratory, Electronics Science and Technology Division, Washington, DC 20007 USA; 2 Sandia National Laboratory, Center for Integrated Nanotechnologies, Albuquerque, NM 87185 USA

## Abstract

By combining optical imaging, Raman spectroscopy, kelvin probe force microscopy (KFPM), and photoemission electron microscopy (PEEM), we show that graphene’s layer orientation, as well as layer thickness, measurably changes the surface potential (Φ). Detailed mapping of variable-thickness, rotationally-faulted graphene films allows us to correlate Φ with specific morphological features. Using KPFM and PEEM we measure ΔΦ up to 39 mV for layers with different twist angles, while ΔΦ ranges from 36–129 mV for different layer thicknesses. The surface potential between different twist angles or layer thicknesses is measured at the KPFM instrument resolution of ≤ 200 nm. The PEEM measured work function of 4.4 eV for graphene is consistent with doping levels on the order of 10^12^cm^−2^. We find that Φ scales linearly with Raman G-peak wavenumber shift (slope = 22.2 mV/cm^−1^) for all layers and twist angles, which is consistent with doping-dependent changes to graphene’s Fermi energy in the ‘high’ doping limit. Our results here emphasize that layer orientation is equally important as layer thickness when designing multilayer two-dimensional systems where surface potential is considered.

## Introduction

Vetting graphene as a candidate for advanced electronics requires examination of both intrinsic and extrinsic influences on its material properties, as well as a nuanced characterization of how graphene responds in different heterogeneous configurations. It is now well documented that graphene’s atomically thin carbon backbone makes it susceptible to influences from its supporting substrate and to any over-layers or adsorbates on its surface^1–3^. As the field of two-dimensional (2D) crystals expands to include stacking layered materials, the relative orientation between layers becomes another important variable. In the simplest case, stacking two graphene layers to form twisted bilayer graphene (TBG) already leads to measureable differences in interlayer screening^4^, optical absorption^5,6^, chiral charge carriers^7^, and chemical reactivity^8^.

As compared to AB-stacked Bernal bilayer graphene, the interlayer electronic coupling in TBG rapidly diminishes as the twist angle (θ) increases above only a few degrees. Once a twist is introduced, a moiré pattern forms from the overlapping lattices. Intriguingly, this moiré structure can itself induce a periodic potential that influences the electronic structure^9,10^, a feature unique to atomically-thin systems. Increasing θ greater than ~20°, the layers are effectively decoupled and the electronic properties are indistinguishable from those in single-layer graphene^11^. Notably, graphene’s small density of states and close interlayer spacing leads to incomplete charge screening, which can sustain a charge imbalance (Δ*n*) between TBG layers with a measured interlayer capacitance around 7 μF/cm^2 ^^4,12^.

To date, variations in graphene’s surface potential (Φ) versus layer thickness (*N*)^13–15^, doping (*n*)^16,17^, and carrier type^18^ have been considered. The combination of extrinsic doping from graphene’s support substrate (typically SiO_2_) paired with interlayer screening helps explain measured differences in Φ with *N*. Density functional calculations^15^ and experiments^16^ show that single layer and bilayer graphene’s work function are tied to its Fermi level (*E*_F_) shift with respect to the Dirac point, while interlayer screening only becomes relevant when more than two layers are present. Since the difference in work function between graphene and, for example, a KPFM tip defines the contact potential (or relative surface potential (ΔΦ)), ΔΦ is also directly influenced by changes in *E*_*F*_ (or n). More broadly, the connection between *n* and Φ has important implications for a variety of systems, from designing low contact barrier electrodes^16,19^ to changing surface wettability^20^. For example, by varying *E*_*F*_ by 300 meV, graphene’s surface energy varies enough to change the water contact angle by as much as 13°^20^.

In this letter, we present the first results mapping the surface potential of graphene layers with varying twist angle and thickness. Importantly, by analyzing the relative surface potential between different regions, we can reduce extrinsic influences of the tip work function, contaminants, feedback, or instrumentation effects^21^, and gain a clearer picture of how the work function varies across the surface. Our findings reveal that twist angle variations can influence Φ by the same magnitude as that found for changes in layer thickness, which reinforces that θ must be carefully considered in designing 2D multilayer systems.

## Results

Graphene films were grown by chemical vapor deposition (CVD) on copper foil ‘enclosures’^22^ at 1030 °C using mixtures of H_2_/CH_4_ and subsequently transferred via wet chemical etching and a PMMA support film to SiO_2_ (100 nm)/Si substrates. We choose graphene formed on the ‘outside’ surface of the Cu-foil enclosures for analysis since multi-layer islands consistently form there^23^. The resulting graphene films range in thickness from 1-layer (1LG) to 6LG and have a random assortment of stacking sequences, with domain sizes on the order of tens of microns. We note that each additional graphene layer grows beneath the continuous first layer^24^, forming an inverse ‘step pyramid’ type structure, which is preserved during the transfer process.

**Figure 1:**
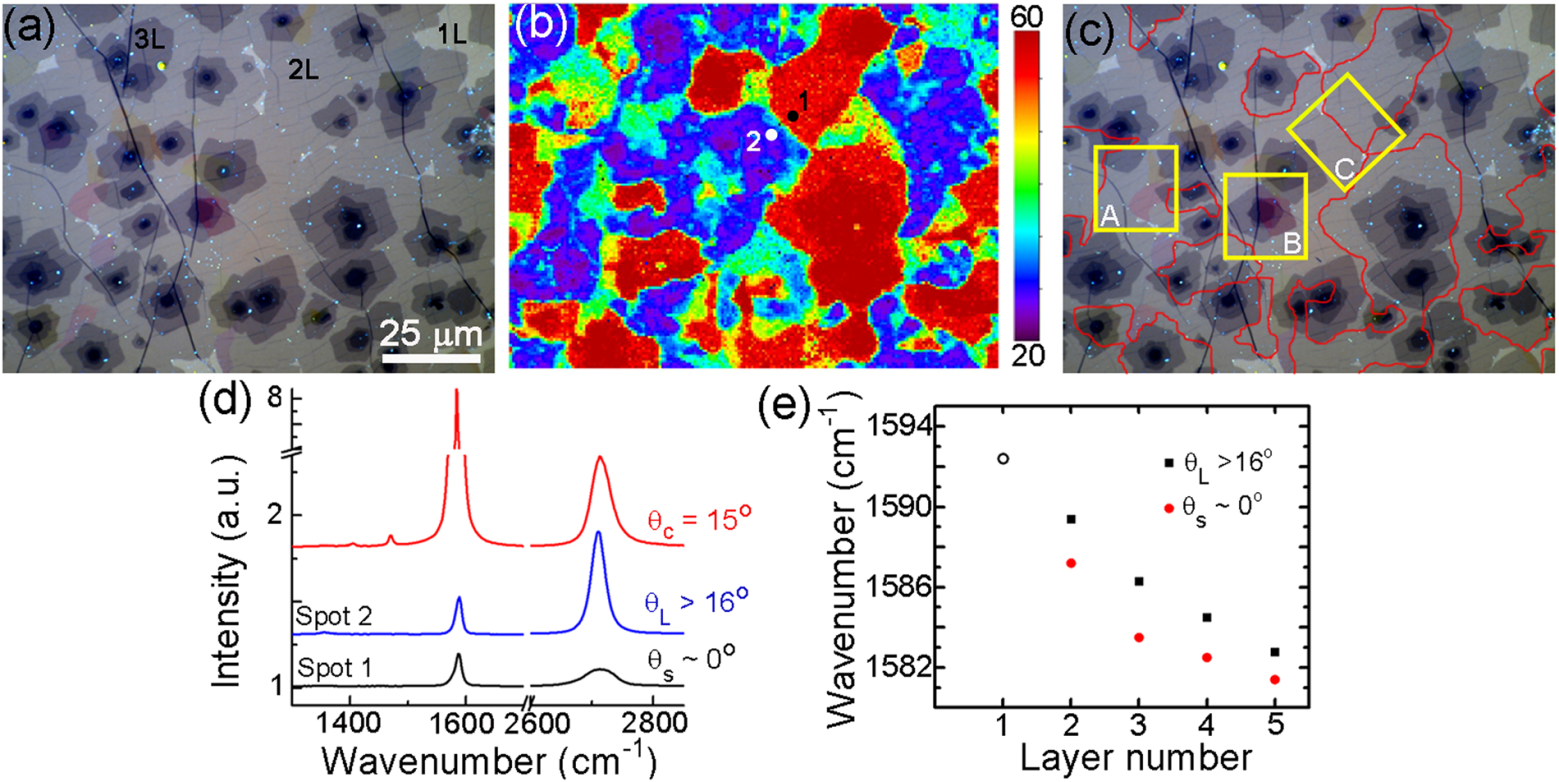
Optical and Raman imaging of multilayer, turbostratic CVD graphene films. (**a**) Optical image of a multilayer CVD graphene film on a SiO_2_ (100 nm)/Si substrate. Labels are shown for thicknesses between 1–3 layers (Note: image look-up table (LUT) adjusted to enhance contrast). (**b**) Raman map (λ = 488 nm) showing the 2D peak (2700 cm^−1^) FWHM from (**a**) (units = cm^−1^). (**c**) Composite image formed by outlining all regions in (**b**) with FWHM ≥50 cm^−1^. The labeled yellow boxes highlight regions mapped out by KPFM. (**d**) Individual Raman spectra from different TBG domains in (**b**). Spectra from spot “1” and “2” in (**b**) are labeled. (**e**) Spatially averaged G-peak position versus layer number for ‘large’ and ‘small’ twist angles in (**b**).

Using a combination of Raman spectroscopy^25,26^ and optical imaging^5,6^, we map out layer thickness (using the quantized nature of graphene optical adsorption) and twist angle domains in the multilayer CVD graphene films (Fig. 1). In particular, there are unique signatures of TBG domains that can be quickly identified. First, white-light imaging of graphene on 100 nm thick SiO_2_ shows the presence of three colored domains (‘blue’, ‘red’, ‘yellow’) that correspond to TBG twist angles of θ_blue_ ≈ 11°, θ_red_ ≈ 13°, θ_yellow_ ≈ 15°^6^. Second, Raman mapping shows variations in the G peak enhancement, as well as the 2D peak full width at half maximum (FWHM), which differentiates between Bernal-stacked (θ = 0°) and turbostratic bilayers (θ > 0°)^25–27^. Figure 1b shows a map of the 2D peak FWHM from the region shown in Fig. 1a. By overlaying the images and outlining all FWHM areas greater than 50 cm^−1^, we have a good approximation of the location of the smallest twist angles and Bernal regions (Fig. 1c)^12,27^. Fig. 1d shows examples of individual spectra from Fig. 1b highlighting a Raman resonant domain (θ_c_ ≈ 15°), a ‘large’ angle domain (θ_L_ > 16°), and a ‘small’ angle domain (θ_s_ ≈ 0°). Finally, Fig. 1e shows the dependence of the G peak position on layer thickness and twist angle, which provides an estimation for doping variations. Given the propensity of ambient H_2_O and O_2_, as well as the underlying SiO_2_ substrate, to hole dope graphene^1^, we assume these films are p-type doped.

From these films, we conduct ambient surface potential measurements using a variation of the Frequency-modulated Kelvin Probe Force Microscopy (FM-KPFM)^28^ technique called PeakForce-KPFM (PF-KPFM; Bruker AFM system). Rather than measuring surface topography and surface potential (Φ) in a one-pass technique, we used a two-pass technique. In the first pass, the surface topography, stiffness, and adhesion are measured in a non-resonant peak-force tapping approach. In the second pass, the surface potential is measured. Unlike conventional FM-KPFM measurements, the tip flies at a fixed user-defined distance above the sample surface (50 nm here), which determines the spatial resolution (~200 nm; see METHODS). By not having the tip touch the surface while measuring the surface potential, one avoids mechanical cross-talk caused by tip-sample interactions that shift the cantilever resonance behavior and affect the sideband detection used in FM-KPFM.

**Figure 2:**
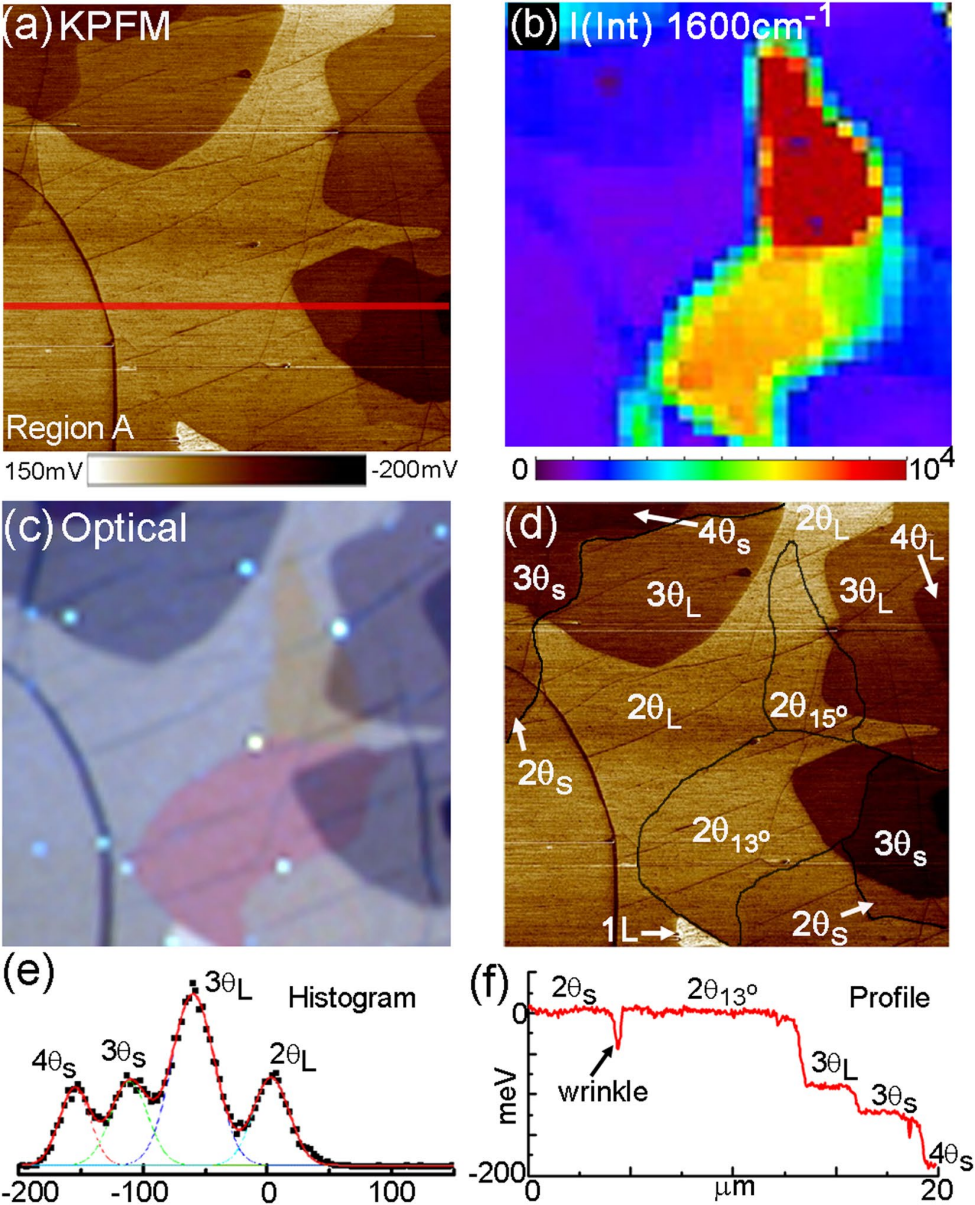
KPFM and optical imaging of multilayer, turbostratic CVD graphene films. (**a**) PF-KPFM image (‘Region A’ in Fig. 1c, image size = 20μm). (**b**) Raman map of the integrated G peak intensity from (**a**) revealing the location of twist domains θ = 15° and θ = 13° (scale bar units = counts). (**c**) Optical microscope image from the same region in (**a**) showing a ‘red’ and ‘yellow’ TBG domain (Note: image look-up table (LUTs) adjusted to increase contrast). (**d**) Labeled PF-KPFM image from (**a**) showing the boundaries of different twist angle domains and layer thicknesses. (**e**) Histogram from the very top part of image (**a**) showing a local distribution of surface potential versus counts (y-axis: arbitrary units; x-axis: mV). The red line is a best-fit using four Gaussian peaks. (**f**) Line profile of the surface potential taken at the red line drawn in (**a**).

**Figure 3:**
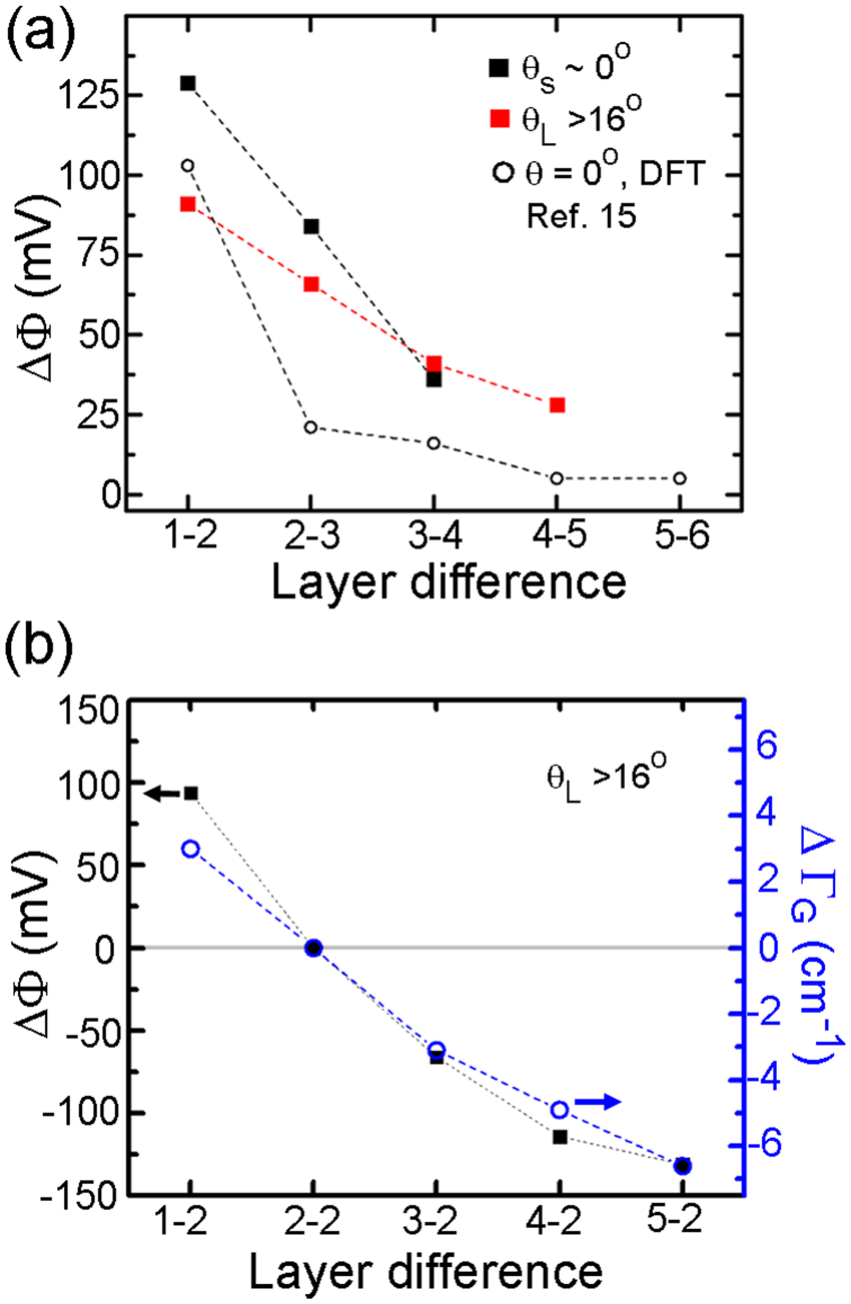
Variations of KPFM surface potentials. (**a**) Plot showing the difference in surface potential (ΔΦ^N^) between graphene layers with different orientations, where on the x-axis ‘1–2’ is the Φ difference between layer 1 and layer 2 for a given twist angle. For example, for θ_L_ domains ΔΦ^1–2^ = 91 mV and ΔΦ^2–3^ = 66 mV. (**b**) Plot comparing the difference in surface potential between twisted bilayer graphene and layers 1–5. The right y-axis plots the difference in Raman G peak position (ΔΓ_G_) between twisted bilayer graphene and layers 1–5.

The PF-KPFM image in Fig. 2a shows a representative example of how Φ varies over a 20 μm area (‘Region A’ in Fig. 1c). Using optical imaging (Figs. 1a and 2c) and Raman spectroscopy (Figs. 1b and 2b), we map out specific twist domains and layer thicknesses (see METHODS), and then directly correlate differences in Φ with these morphological features (Fig. 3). Figure 2d shows a composite PF-KPFM image produced using this approach and identifies four twist domains and four layer thicknesses (*e*.*g*. ‘3θ_s_’ = 3LG with a small twist angle (θ ≈ 0°); ‘2θ_13_’ = 2LG with θ = 13°; ‘2θ_L_’ = 2LG with large twist angle (θ > 16°)). The image signal-to-noise ratio is sufficiently large that we can use either pixel histograms (Fig. 2e) or line profiles (Fig. 2f) to quantify differences in Φ over the surface. When line profiles are taken perpendicular to step or twist boundaries, the transition step widths are measured at the instrument resolution of 200 nm.

The relative surface potential difference (ΔΦ) between sequentially increasing layer number [N − (N + 1)] is shown in Fig. 3a, where the stacking orientation here consists of layers with θ_L_ > 16° (red squares) or θ_s_ ~ 0° (black squares). For example, the relative potential difference between 1LG and 2LG (written as ΔΦ^1−2^) for θ_L_ is 91 mV, whereas ΔΦ^1−2^ for θ_s_ is 129 mV. Notably, other works have shown measured values for epitaxial or exfoliated graphenes at ΔΦ^1–2^ = 135 mV^13^, 120 mV^16^, 110 mV^28^, or 66 mV^15^, though the layer orientations were not measured in these reports. For comparison, in Fig. 3a we also show reported DFT calculations^15^ of ΔΦ for Bernal stacked graphene layers (θ = 0°). We note that Φ is sensitive to both carrier concentration and band structure (discussed below). To further visualize the correlation between Φ and *n*, in Fig. 3b we plot both the surface potential referenced to TBG (ΔΦ^N-2^) for θ_L_ domains versus the relative difference for the G-peak position (ΔΓ_G_) as referenced relative to TBG. This comparison reveals a qualitative relationship between the variables ΔΦ and ΔΓ_G_.

## Discussion

Variations in graphene’s Fermi energy, or charge carrier density, induce shifts in the Raman G peak position (Γ_G_), as seen in Fig. 1e. Fundamentally, deviations in Γ_G_ are a result of electron-phonon coupling and changes in bond stiffness^27,29^. When the Fermi level is at the Dirac point (*E*_D_), the G-peak position is close to 1580 cm^−2^ and increases symmetrically for hole or electron doping^27,29^. In our sample geometry, the top most graphene layer is a continuous single-layer film and on the length scales considered here, uniformly exposed to ambient dopants (e.g., O_2_/H_2_O). The additional buried layers are protected from the ambient and are instead directly influenced by the nominally spatially homogeneous (at this length scale) trapped charges in the underlying SiO_2_. Thus, lateral differences in *n* across the film should directly correlate to how multilayer graphene regions with θ_L_ or θ_s_ screen charges from their ‘top’ and ‘bottom’ surfaces.

From the Raman data shown in Fig. 1e we can correlate how *n* varies with morphological features of our samples (θ and *N*). For all layers measured here, we find large twist angle domains (θ_L_ > 16°) have higher doping concentrations compared to small twist angle domains (Fig. 1e). This correlation is a consequence of less effective screening for θ_L_ than θ_S_. In addition, the trend in G-peak position for θ_L_ regions varies approximately linearly with *N*. This linear dependence of G-peak position with θ_L_ regions is consistent with the fact that: (i) for single-layer graphene at ‘high’ carrier densities (*n*>~ ± 0.5 × 10^12^ cm^−2^) the G-peak energy increases linearly with *E*_*F*_ (or *n*)^29,30^ and (ii) θ_L_ multilayers behave as an ensemble of independent single-layer graphene despite their N > 1 thicknesses. Notably, our Raman measurements probe all layers here and provide an average doping level for the multilayer stack. Given the linear change in G-peak position with increasing *N* and the fact that θ_L_ multilayers behave essentially as independent graphene layers, we surmise that the carrier concentration per unit area is distributed over the multilayer stack. A linear regression fit for θ_L_ data in Fig. 1e yields a slope of *m* = −2.4 cm^−1^/layer. The increased interlayer coupling/screening for θ_s_ multilayers results in a lower average doping level across all *N* and is most likely a result from screening that is nonlinear to the field amplitude^31,32^, a result of the different band structures between θ_L_ and θ_s_ multilayers.

To connect our spatially varying doping concentration with PF-KPFM measurements, we briefly review the relationship between *E*_F_, *n* and Φ. Using electrostatic gating to control *n* between approximately 1.5 × 10^12^cm^−2^ to −2.5 × 10^12^cm^−2^, Yu *et al*.^16^. Showed their graphene work function data could be fit using the relationship ${E}_{F}\propto \sqrt{n}$ for 1LG and ${E}_{F}\propto n$ for 2LG (θ = 0°). This relationship was further supported by DFT calculations of Bernal-stacked graphene layers having charge doping on their top or bottom surfaces^15^. To visualize how Φ varies with the morphological features of our films, in Fig. 3a we plot the relative difference in Φ for sequentially increasing layers and compare to Φ found from DFT calculations^15^. From this plot several features are apparent: (i) the surface potential drop (ΔΦ) between layers with sequentially increasing *N* monotonically decreases; (ii) the rate of decrease for ΔΦ is larger for θ_s_ compared to θ_L_; and (iii) the trends for both θ_s_ and θ_L_ are approximately linear, whereas DFT calculations shown a large initial drop followed by a slowly decreasing potential. We note that ΔΦ^1–2^ for both our measurements and DFT calculations are similar (within 20 mV), which is reasonable since both systems have approximately the same initial doping concentration (~5 × 10^12^cm^−2^).

For intrinsic graphene (no doping) there is no difference in Φ between 1LG and 2LG, while at low finite doping Φ varies with *E*_F_ as described above^15,16^. In our experiments we cannot extrinsically tune *n*, but we do have a range of *n* due to the relatively homogeneous doping from ambient and substrate with varying *N* and θ. In addition, these samples are in the ‘high-doping’ limit where Φ trends approximately linearly for both single and multilayers. This linear correlation can been seen in Fig. 3b, where we simultaneously plot the relative potential difference for all θ_L_ layers as compared to TBG (ΔΦ^N-2^), as well as relative Raman G peak positions, ΔΓ_G_ (a function of *n*), between TBG versus layer number. On a linear scale, both ΔΦ and ΔΓ_G_ have a monotonically decreasing trend with similar shape, which qualitatively suggests a proportionality constant between them. This scaling is notable since the PF-KPFM measurements are uniquely sensitive to surface variations as compared to Raman spectroscopy, which equally probes all layers in the film.

**Figure 4:**
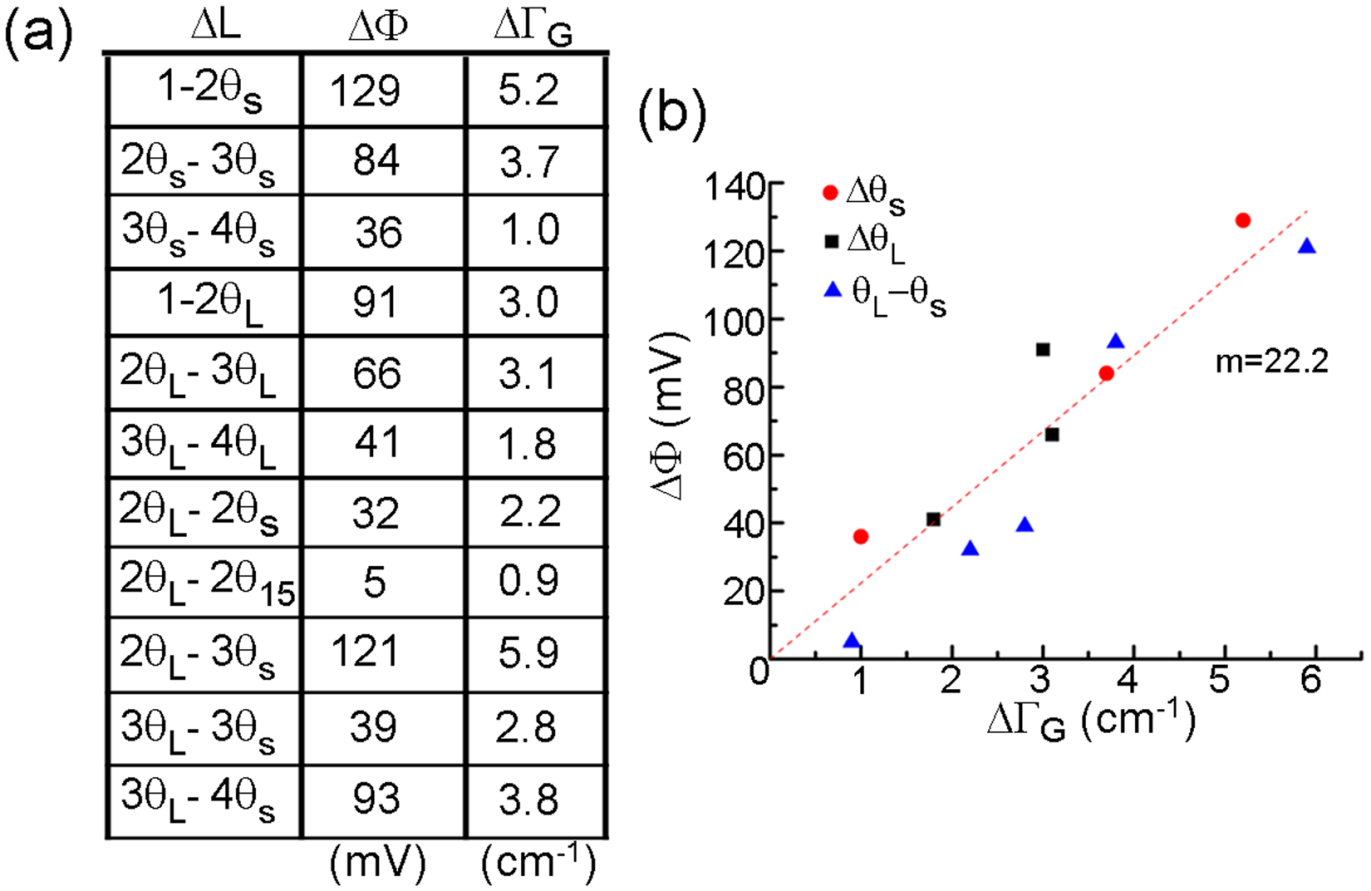
KPFM potential differences between specific layers and twist domains. (**a**) Table identifying the corresponding change in surface potential (ΔΦ) and Raman G peak wavenumber difference (ΔW) for given layer thickness and twist angle differences (ΔL). (**b**) Plot of surface potential difference versus ΔΓ_G_ from (**a**). The best-fit linear regression through zero for ALL data points is shown with corresponding slope (*m* = 22.2 mV/cm^−1^).

To further assess the relationship between doping and surface potential, in Fig. 4 we list and plot ΔΦ and ΔΓ_G_ between all measured configurations of *N* and θ domains in our samples. The table in Fig. 4a was populated using only the relative difference between directly neighboring *N* and θ domains from a given PF-KPFM image. Plotting this data in Fig. 4b, we see no apparent difference in the trend of Φ versus Γ for different θ. This is not surprising in light of the fact that these samples are in the high doping limit for both 1LG and 2LG, where *E*_*F*_ is approximately linear with Γ_G_.

Given our films are in the high doping limit, we apply a linear fit to all data in Fig. 4b and we find a proportionality constant of 22.2 mV/cm^−1^. Using the proportionality constants between different variables (e.g., *E*_*F*_, *n*, and Γ_G_) for single-layer graphene, we can approximate the doping levels required to change the surface potential in our films. For 1LG in the high-doping limit, the proportionality between Γ_G_ versus *E*_F_ equals −0.04 cm^−1^/meV (negative sign for holes, positive for electrons)^29^, resulting in *n* = 1 × 10^12^ cm^−2^ carriers for *E*_F_−*E*_D_ = 40.3 meV^30^. Taken together with our data, we approximate the relation between ΔΦ versus *n* at 35.8 mV per 1 × 10^12^ cm^−2^ carriers. Importantly, this comparison reveals that ΔΦ between different twist angles can have the same magnitude as that for ΔΦ between different layer thicknesses, which appears to be driven by how a specific morphological feature (such as *N* or θ) screens charge.

**Figure 5:**
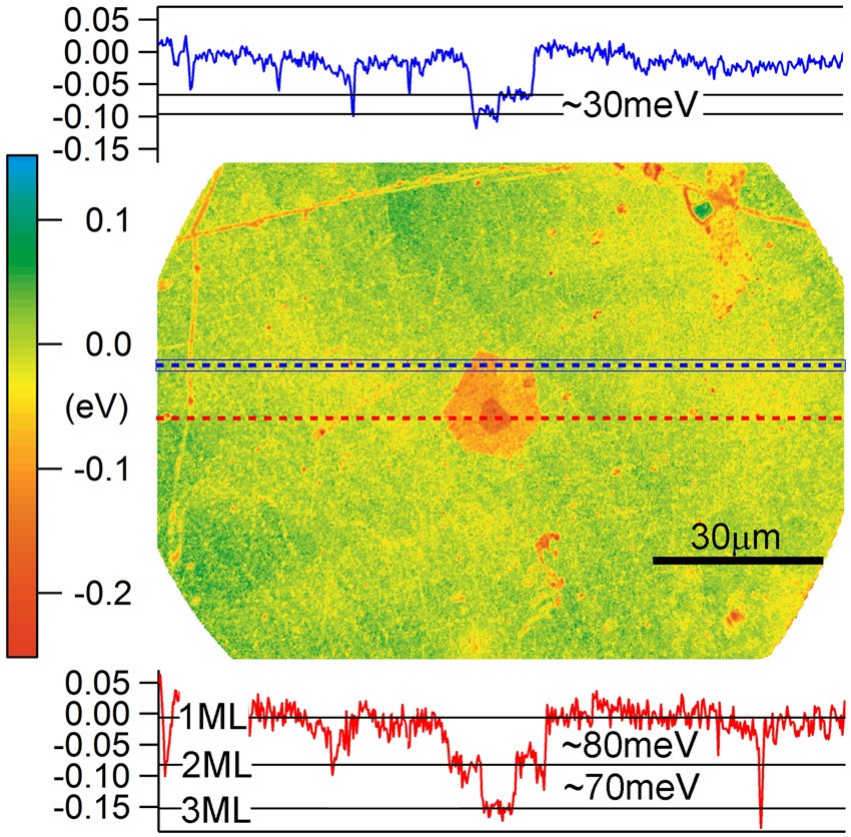
PEEM analysis of twisted graphene films. The work function map acquired from the secondary electron cutoff of the photoemission spectra using PEEM, and calibrated using the photoemission yield measurement of 1LG. (insets) Line profiles of the work function along the red and blue dash lines.

To independently confirm the surface potential variation as a function of graphene morphology, we conducted spectroscopic Photoemission Electron Microscopy (PEEM) analysis of multilayer graphene films. Figure 5a shows the work function map of a 1LG film with a smaller island containing 2LG (skewed hexagon in light orange) and a 3LG island (smaller red area at the center). The assignments of island thicknesses are based on optical micrographs of the sample (e.g., Fig. 1a).

Consistent with the PF-KPFM measurement shown in Figs. 2a and 2f, areas of thicker graphene in Fig. 5a exhibit lower work function. The quantitative difference of the work function between 1LG and the multilayer island is illustrated in the line profiles along the red and blue dotted lines in Fig. 5. The profiles here show ΔΦ^1–2^ ≈ 80 meV and ΔΦ^2–3^ ≈ 70 meV, which agrees with the surface potential difference determined for θ_L_ using PF-KPFM (Fig. 3a). A close analysis of the work function map also reveals a potential difference within the 2LG island (upper-left quadrant). In this 2LG region there is an approximate 30 meV potential step, as shown in the line profile along the blue dotted line (Fig. 5c). Given the rotationally faulted nature of our multilayer CVD graphene (e.g. Figs. 1 and 2), we assign this ΔΦ to the boundary between a 2θ_S_ and 2θ_L_ domain. With this assignment, the photoemission data and PF-KPFM data for ΔΦ between 2θ_S_ and 2θ_L_ domains are in excellent agreement.

Since the work function of thin graphite films is mainly determined by variation of the Fermi energy with respect to the Dirac point energy as a function of doping^15^, one might expect that the doping concentration could be estimated from the work function. This is not the case, however, because of the limited consensus on the work function of graphene. The reported work function values of 1LG supported on SiO_2_ (with no gate bias) vary significantly from 4.2 eV^15,33^ to 5.1 eV^34^, with a commonly accepted range of 4.5–4.6 eV^16,35^. Given this relatively wide scatter of reported work function values, the value of 4.4 eV for 1LG measured here via photoemission is within a reasonable range for a carrier concentration of 10^12^cm^−2^ (estimated from Γ_G_).

In conclusion, we quantitatively assess the surface potential of twisted graphene layers as measured by PeakForce Kelvin Probe Force Microscopy (PF-KPFM) and Photoemission Electron Microscopy (PEEM). By combining optical microscopy, Raman spectroscopy and PF-KFPM measurements of the same graphene regions, we find a linear correlation (slope = 22.2 mV/cm^−2^) between the surface potential and G-peak Raman shift for regions of different thickness (*N*) and twist angle (θ). Using this correlation, we estimate the proportionality between graphene’s surface potential and doping concentration at 35.8 mV per 1 × 10^12^ cm^−2^ carriers. Furthermore, we find that the rate at which the surface potential decreases for increasing layer number is larger for ‘small’ twist angles (θ_s_ ≈ 0°) as compared to ‘large’ twist angles (θ_L_ > 16°), which is consistent with the stronger interlayer interaction for small twist angle samples. Finally, using PEEM we independently confirm the surface potential differences for different morphological features (*N* and θ) in multilayer CVD graphene. Together, these results reinforce the notion that layer orientation, or twist angle, is another critical variable to account for when designing 2D multilayer systems.

## Methods

### Raman Spectroscopy

Raman measurements were performed using a confocal geometry. Dichroic beam splitters were used to reflect single-mode 488 nm laser light onto the excitation/detection optical axis. A 100× microscope objective (NA = 0.65) focused the laser (spot ≈0.4 μm) onto the sample and gathered Raman scattered light for detection. The Raman scattered photons were dispersed in a half-meter Acton Sp-2500 spectrometer and were detected using a Princeton Instruments CCD array (Spec-10:400BR back-thinned, deep-depleted array). Optical images were acquired using a microscope with a Nikon DS-Ri2 high-definition color camera (16.25 megapixel).

### Twist angle assessment

For multilayer graphene >2 L, we can say with confidence which regions have all layers with relative twist angles θ > 16° or θ = 0°. The label “θ > 16°” (or “θ = 0°”) here means that directly neighboring layers in the ML stack have twist angles at least >16° (or θ = 0°). We make these assignments using optical inspection (graphene contrast is quantized per layer) and raman analysis of the G and 2D peaks. The raman spectra and optical micrographs will be a convolution of the couplings between every layer in the ML stack. The stronger interlayer coupling for low twist angles (θ < 5°) readily shows up as broadening in the Raman 2D peak FWHM, while the optically resonant domains readily show up in the optical micrographs. As such, we can discern between ‘rotationally pure’ regions with the bounds of θ = 0° (i.e. all strongly coupled) or θ > 16° (i.e., all fully decoupled). Since we are analyzing a maximum of 5-layers or less in this work, convoluted mixtures of twist angles can be identified. In Fig. 1e we only show raman data for regions that have all layers with θ = 0° (Bernal stacking) or θ > 16° (fully decoupled layers).

### AFM measurements

AFM measurements were performed on a Bruker Dimension Fastscan with ScanAsyst^TM^ microscope using an Icon scan head. Measurements were taken in air while enclosed in the systems’ quiet box. While the KPFM data presented here was acquired from different regions of the same sample to reduce variability, we have confirmed that different graphene growths produce comparable results for ΔΦ between different twist angles and layer thicknesses.

KPFM Sensitivity- Because the tapping during the topography measurement pass is not done at the mechanical resonance frequency of the tip, the *Q* of the cantilever’s resonance and the spring constant (*k*) of the cantilever can be separately chosen to optimize the surface potential measurement. TappingMode requires the cantilever to have a sufficiently high *k* to overcome capillary forces from water layers that are often present on sample surfaces. It also requires the *Q* to be not so large as to limit TappingMode bandwidth. PeakForce Tapping removes these limitations, allowing the use of cantilevers with a much smaller spring constant and larger *Q*, enabling room for probe optimization. The PFQNE-AU probes used in this work are twice as sensitive as the commonly used SCM-PIT probes. Probes with higher *Q*/*k* ratio can be designed to further improve PeakForce KPFM sensitivity^36^.

Spatial resolution- As outlined in the instrument Application Notes^36^, “for FM-KPFM, when the tip is lifted 5 nm above the surface, half of the signal is gathered from up to 15 nm above the tip end, corresponding to a diameter of 12 nm. This suggests a possible resolution of 10 nm may be achieved with FM-KPFM. The contact potential difference (CPD) information collected is local to the area right underneath the tip affording credible accuracy. Lifting the tip higher to 50 nm above the surface [as used in this work], the spatial resolution decays to around 200 nm, which demonstrates the sharp dependence of spatial resolution on probe-sample distance.”

### Photoemission Spectroscopy

Photoemission spectra were acquired using a photon energy of 6.53 eV. A work function map with a relative energy scale was created by fitting the secondary electron cutoff of the photoemission spectra pixel-by-pixel for the entire imaged area. The relative energy scale was calibrated using the work function of 1LG, determined from photoemission yield measurements acquired by recording the photoemission intensity as a function of photon energy (for details of the data acquisition and processing, see ref.^37^). The photoemission yield measurement itself did not yield sufficient statistics to bear a work function map of the same area due to the low photoemission intensity. We note that the sample showed no sign of charging because of the continuous coverage of graphene films across the sample^38^. In addition, the use of a deep ultraviolet light for PEEM measurement helped mitigate sample charging as demonstrated in PEEM of transition metal dichalcogenide semiconductors on SiO_2_^39^.

Surface contaminants, such as PMMA residues, typically show up as non-uniformities in the photoemission intensity and coincide with a smearing of the vacuum level (*E*_vac_) and valence band edges in PES. The energy scale of *E*_vac_ smearing due to residues, and hence the smearing of the relative work function, is on the order of 100 s of meV (~350 meV). By comparing optical microscopy images with PEEM images and performing spectroscopic PEEM measurements of the graphene samples studied here, we avoided areas with non-uniformities in optical or PEEM intensities. As a result, work function variations in graphene were resolved with PEEM on an energy scale (10s meV), similar to what was observed with as-grown MoS_2_^37^. Graphene work function values (4.4 eV for 1 L graphene) measured here with PEEM are consistent with several prior works^15,16,35^.
